# Effectiveness of bovine-derived xenograft versus bioactive glass with periodontally accelerated osteogenic orthodontics in adults: a randomized, controlled clinical trial

**DOI:** 10.1186/s12903-016-0321-x

**Published:** 2016-11-30

**Authors:** Maha A. Bahammam

**Affiliations:** Department of Periodontology, Faculty of Dentistry, King Abdulaziz University, P. O. Box 80209, Jeddah, 21589 Kingdom of Saudi Arabia

**Keywords:** Alveolar bone density, Bone grafts, Corticotomy-assisted orthodontic treatment, Root resorption

## Abstract

**Background:**

Periodontally accelerated osteogenic orthodontics (PAOO) combines periodontal therapy with orthodontic therapy, which minimises treatment time. This study compared the effectiveness of a bovine-derived xenograft with that of bioactive glass when combined with PAOO for the treatment of adult patients with moderate crowding of the teeth.

**Methods:**

In this prospective, single-masked clinical trial, 33 orthodontic patients (20 women, 13 men; mean age 21.2 ± 1.43 [18 − 27] years), were randomly allocated to one of three groups. Group 1 underwent a modified corticotomy technique on the labial side only, whereas group 2 was treated with the same technique combined with PAOO using a bovine-derived xenograft and group 3 was treated in the same way but combining PAOO with bioactive glass. The total treatment duration was recorded from the start of active orthodontic treatment, immediately after corticotomy, and at the time of debonding. Probing depth was evaluated clinically and bone density and root length were evaluated radiographically on the day of surgery (baseline, T1), post-treatment at debonding (T2), and 9 months post-treatment (T3).

**Results:**

The duration of orthodontic treatment was markedly reduced to an average of 11.4 ± 0.14 weeks in all groups. All probing depths were < 3 mm, the interdental papillae were well preserved, there was no loss of tooth vitality, and there was no evidence of significant apical root resorption at any time interval. All groups showed a decrease in mean bone density at T2 followed by an increase at T3. The net percentage change that occurred between baseline and 9 months post-treatment was significantly different between the three groups. Groups 2 and 3, where grafts were incorporated, demonstrated a statistically significant greater increase in bone density than group 1 at T3.

**Conclusion:**

Combination of orthodontic treatment and periodontal surgery is an effective treatment for adult patients that decreases the duration of active treatment and reduces the risk of root resorption. Use of a bovine-derived xenograft with modified corticotomy provided superior benefits in terms of increased bone density than did the use of bioactive glass.

**Trial registration:**

The study was retrospectively registered at ClinicalTrials.gov under Clinical Trial Registration Number: NCT02796911.

## Background

There has been a significant increase in the number of adult patients seeking orthodontic treatment [1]. The average duration of routine orthodontic treatment for adults is considerably longer than for adolescent patients, ranging from 18.7 to 31 months, and even more time is required for extraction cases [2, 3]. Adult patients are also more vulnerable to root resorption and periodontal pathologies during or after active orthodontic treatment, given their narrow, aplastic, poorly vascularised periodontal membrane, and alveolar bone morphology [1, 4]. Hence, it is imperative to modify treatment modalities to reduce the treatment time and achieve optimal clinical results in adults.

Corticotomy and osteotomy have been used in orthodontics primarily to resolve crowding of teeth as rapidly as possible. In published case reports, moderate or severe crowding has been treated without extraction by corticotomy/osteotomy-assisted orthodontics, and in shorter time periods [5, 6]. It has been reported that this approach was efficient for reducing the treatment time to as little as a quarter of that usually required for conventional orthodontics [5, 7–9]. Additional applications for this technique have reportedly facilitated several orthodontic modalities in a short period of time, with greater post-orthodontic stability [7, 10].

A temporary stage of remodelling of localised soft and hard tissue, termed the “regional acceleratory phenomenon” (RAP), results in rebuilding of the sites injured in corticotomy/osteotomy to a normal state by recruitment of osteoclasts and osteoblasts via local intercellular mediator mechanisms that involve precursors, supporting cells, blood capillaries, and lymph. RAP begins within a few days of the insult, typically peaks at 1 − 2 months, and may take as long as 2 years to subside completely [11, 12].

Periodontally accelerated osteogenic orthodontics (PAOO) is a technique involving combination of a selective decortication-facilitated orthodontic technique and alveolar augmentation. Thus, teeth can be moved 2 − 3 times further in one third to one quarter of the time required by traditional orthodontic therapy. Moreover, PAOO provides an increased net alveolar volume after orthodontic treatment and is associated with low morbidity [9, 13]. The greater alveolar volume eliminates bony dehiscence and fenestrations under most circumstances [5]. Likewise, the additional alveolar bone width may be the cause of the enhanced long-term orthodontic stability noted with this technique [13, 14].

Hong et al. conducted a study in 2011 using a Beagle animal model for histologic assessment of the biological effects of PAOO. In the group that underwent corticotomy, no osteoclasts were found on the cementum or dentin, although numerous osteoclasts were detected around the alveolar bone facing the palatal side of the retracted incisor. In addition, a more intact periodontal ligament space of consistent thickness was noted between the surrounding alveolar bone and the root surface. The compression side of the retracted incisor showed no evidence of root resorption [7, 15].

Interestingly, current therapies in both traumatology and tissue regeneration dentistry are based on the use of artificial or natural materials, which produce stimulating signals that trigger physiological regeneration, a process that depends on three mechanisms: osteogenesis, osteoinduction, and osteoconduction [16]. Xenografts and bioactive glass offer such alternatives. A number of previous studies have demonstrated the effectiveness of xenografts as osteoconductive matrices [17–21]. On the other hand, bioactive glass is an alloplastic bone graft and a possible choice for alveolar augmentation in corticotomy-assisted orthodontic treatment (CAOT) [16–25]. Results from clinical studies have indicated that treatment of intrabony defects with bone grafts alone may result in improved clinical outcomes, as evidenced by improvements in probing depth (PD) and clinical attachment [23].

In spite of the advantages of PAOO, assessment of this technique remains challenging in clinical trials, and evidence of the success of these grafts has been evaluated only in a few case reports [5, 8, 26, 27]. Accordingly, the purpose of this study was to evaluate the effectiveness of a bovine-derived xenograft versus that of bioactive glass, using a modified corticotomy procedure, in the orthodontic treatment of adult patients with moderate crowding.

## Methods

### Sample

The study protocol was approved by the ethics committee of King Abdulaziz University in accordance with the guidelines published by the CONSORT group and the World Medical Association’s Declaration of Helsinki, (proposal number 029-16). All research procedures were explained to the patients, who provided signed consent to participate in the study. The study was retrospectively registered at ClinicslTrials.gov under Clinical Trial Registration No. (NCT02796911).

Thirty-three adult orthodontic patients (23 female, 10 male) of mean age 21.2 ± 1.43 (range 18 − 27) years were included in this randomised, prospective, single-masked trial that was conducted between February 2015 and March 2016. The study participants were selected from patients seeking orthodontic treatment at the outpatient clinic in the Orthodontic Department, Faculty of Dentistry, King Abdulaziz University. The criteria for inclusion in the study were as follows: good oral hygiene and systemic health; PD values (measured as the distance from the bottom of the sulcus to the most apical portion of the gingival margin) not exceeding 3 mm; no radiographic evidence of bone loss; no regular administration of any medication that affects bone metabolism, such as prolonged use of corticosteroids, bisphosphonates, or non-steroidal anti-inflammatory drugs; moderate crowding of the lower anterior teeth only, ranging from 4 − 5 mm; and no previous orthodontic treatment.

Cephalometric analyses were performed to limit patient selection to those with a Class I skeletal pattern and a favourable mandibular incisor plane angle. Orthodontic study cast analyses were used to limit the selection to cases of moderate crowding. Standardised digital periapical radiographs from the left mandibular canine to the right mandibular canine were used and the exposure parameters were fixed for all patients, both initially and during the follow-up period.

Each patient was randomly assigned to one of three groups, masked with respect to assignment and each containing 11 patients. Group 1 (seven women, four men) was treated with a modified technique of CAOT alone, group 2 (six women, five men) was treated with CAOT combined with a bovine-derived xenograft (Bio-Oss, Geistlich, Princeton, NJ, USA), and group 3 (seven women, four men) was treated with CAOT combined with bioactive glass (GlassBone, NORAKER, Villeurbanne, France). Randomisation was performed using a commercially available computer software package (NCSS-PASS, Number Cruncher Statistical Systems, Kaysville, UT, USA).

The proposed treatment procedures along with their advantages and disadvantages were explained in detail to all patients and informed consent was obtained. Initial prophylaxis and periodontal therapy, consisting of full mouth scaling utilising both hand and ultrasonic instruments, were performed under local anaesthesia.

Each participant received orthodontic treatment with a mandibular fixed appliance inserted during the week preceding surgery. In accordance with the treatment plan, the standardised treatment protocol used a pre-adjusted appliance that included direct bond brackets (Roth Prescription) 0.022" × 0.028" in size (Integra Brackets, Rocky Mountain Orthodontics Inc, Denver, CO, USA) from the right mandibular second premolar to the left mandibular second premolar, using a chemical cure orthodontic adhesive (Transbond XT; 3 M Unitek, Monrovia, CA, USA) and banding of the mandibular first molars (Rocky Mountain Orthodontics Inc). The appliance was not activated presurgically. Orthodontic tooth movement was started 2 weeks after the corticotomy procedure; the interval allowed between routine orthodontic adjustments was 2 weeks, which is in line with previous recommendations [5, 9, 27].

During orthodontic treatment, the mandibular arch was initially levelled and aligned using nickel titanium arch wires of increasing size (0.012", 0.014", 0.016", and 0.018"). Thereafter, rectangular stainless steel wires (0.016" × 0.022") were placed. Stainless steel arch wires up to size 0.019" × 0.025" were used for finishing. During the active tooth movement phase, patients were assessed by the periodontist at monthly intervals.

### Periodontal surgical procedure

**Fig. 1:**
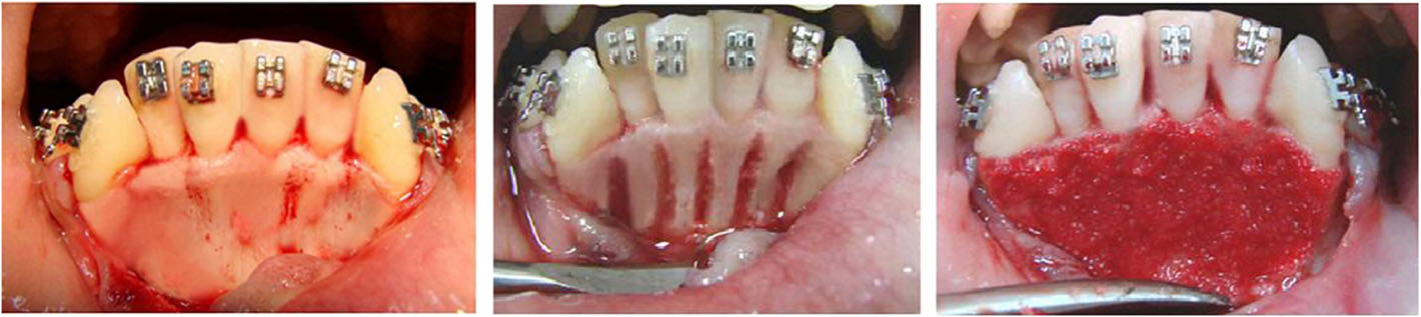
Surgical procedures

The PAOO technique used for the three groups in the current study was performed under local anaesthesia and is a modification of the basic corticotomy technique described by Wilcko et al. [8, 9, 27]. Intra-crevicular full-thickness flaps were reflected labially only from the distal surface of the lower right canine to the distal surface of the lower left canine while preserving the interdental papilla. The labial flap was reflected beyond the apices of the lower anterior teeth. Selective alveolar decortication was then performed in the form of vertical grooves through the labial cortical plate of bone, using a small round stainless steel surgical bur (number 2) in a low-speed hand piece, under copious irrigation, and extended through the entire thickness of the cortical plate, barely reaching the medullary bone. The vertical grooves started 1 − 2 mm below the alveolar crest and extended 1 − 2 mm below the apices of the teeth (Fig. 1). The lingual flap was not elevated and no horizontal subapical cuts were performed [28].

In groups 2 and 3, after completion of the corticotomy procedure, a bovine xenograft and/or bioactive glass were mixed with blood from the surgical site in a sterile Dappen dish until a sandy consistency was obtained. The resulting coagulum was transferred in increments and applied directly over the decorticated areas (Fig. 1). Next, the flap was carefully repositioned at the original pre-surgical site and sutured with nonresorbable 4-0 silk using the interrupted technique [23, 29].

**Fig. 2:**
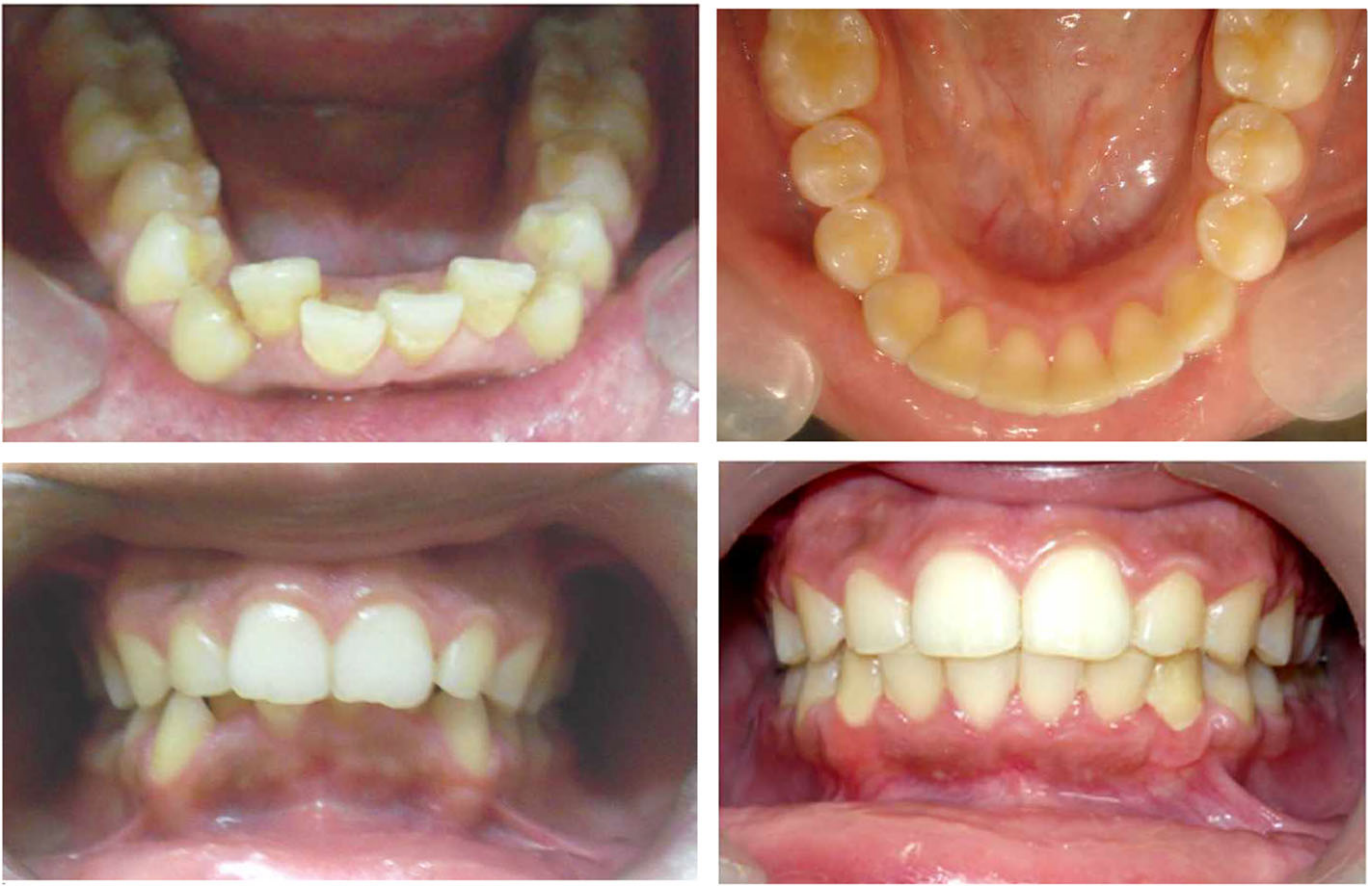
Occlusal and frontal Pre and post treatment intraoral pictures

All patients were provided with oral hygiene instructions and closely monitored to prevent inflammation of the gingival tissues. Antibiotic, diuretic, and analgesic agents were prescribed for 7 days. The efforts to control plaque formation were augmented by antiseptic mouthwash (0.12% chlorhexidine gluconate, for 1 min twice daily for 2 weeks), and the sutures were removed after 10 days. Figure 2 shows representative intraoral images taken before and after treatment.

### Data analysis

The total duration of active orthodontic treatment was estimated in weeks for the three study groups from the time of starting active orthodontic treatment, immediately following the corticotomy procedure, to the time of debonding.

Clinical and radiographic parameters were recorded on the day of surgery (T1), post-treatment (at time of debonding) (T2), and 9 months post-treatment (T3) for all three groups. PD measurements were taken using a William’s probe 26 from the gingival margin to the base of the sulcus and recorded to the nearest millimetre. Six readings for each tooth were recorded (mesial, distal, and midpoint for both the labial and lingual surfaces).

Radiographic measurements were obtained from standardised digital periapical radiographs from the left mandibular canine to the right mandibular canine using Digora system software (Orion Corp, Sordex Medical System, Helsinki, Finland). The radiographic images for each patient were saved and analysed to record bone density and root length, and the mean of the readings was calculated (Fig. 2).

To measure the bone density, the mean grey value in each region of interest was calculated (256 grey levels of colour resolution) by assigning the grey value of 0 to black, and a grey value of 256 to white. Linear density was measured by drawing a line parallel to the root surface, extended from the apex of the alveolar crest to the level of the apex of the root. A line was drawn midway between every two lower anterior teeth. Five lines were drawn (between the lower right canine and lateral incisor, between the lower right lateral incisor and central incisor, between the two lower central incisors, between the lower left lateral incisor and central incisor, and between the lower left canine and lateral incisor).

The grey level along each line was recorded at the beginning, middle, and end of the line, and the average of the three readings was calculated to obtain the mean average density (grey level) along this line. The mean value of the readings of the five lines was calculated to present the density value.

The root length was assessed by measuring the distance between the cemento-enamel junction (as a reference point) and the apex of the root in millimetres.

One operator (MB) performed all the surgeries and was informed of group assignment after corticotomy just before placing the graft material. Another calibrated operator, who was not involved in the study, performed all the clinical and radiographic measurements without knowledge of group assignment. Intra-examiner calibration was evaluated before starting the study by examination of 30 sites on two separate occasions 48 h apart. Calibration was only accepted if 90% of the readings could be reproduced within a 1 mm difference.

The primary outcome of the study was duration of orthodontic treatment from baseline to the time of debonding. Secondary outcomes included changes in PD, bone density, and root length up to 9 months post-treatment.

### Statistical analysis

The collected data were tabulated and statistically analysed using Statistical Package for the Social Sciences version 20 (IBM Corp., Armonk, NY USA). Descriptive statistics (mean, standard deviation, range) are presented. One-way analysis of variance was used to compare data between the three groups, and a post-hoc (Tukey’s) test was used for pair-wise comparisons between the groups when analysis of variance test were significant. The Student’s *t*-test was used to compare the mean differences within each group for each time interval.

## Results

**Fig. 3:**
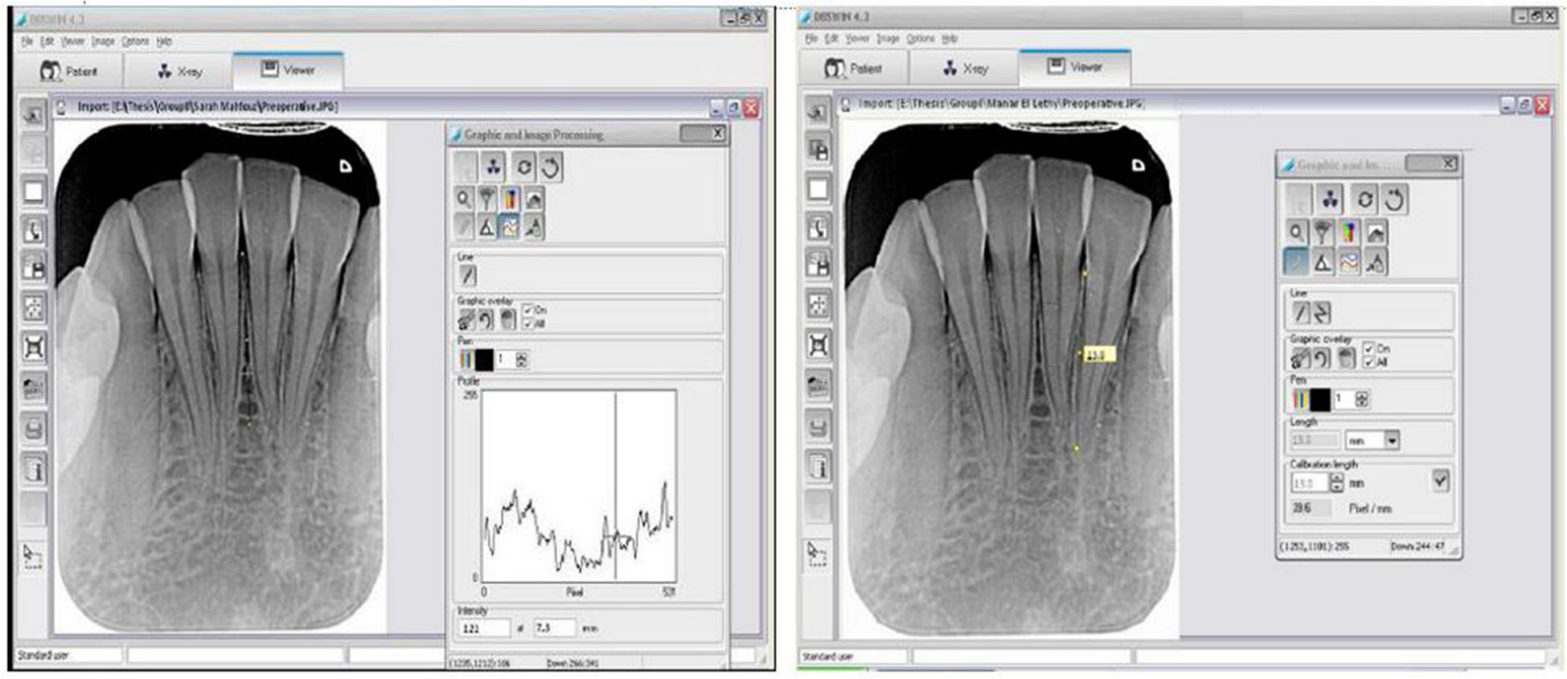
Radiographic assessment

The total treatment time was calculated in weeks from the time of activation of the orthodontic appliance immediately following the corticotomy procedure to the time of debracketing. The treatment duration for patients in all groups ranged from 12 to 20 weeks, with a mean of 15 weeks for group 1, 16.8 weeks for group 2, and 14.4 weeks for group 3. Figure 3 shows representative intraoral images before and after treatment.

### Clinical parameters

#### Probing depth

There was no significant difference in PD at baseline between the three groups (*P* > 0.05; Table 1). Within each group, there was a significant difference between the participants in terms of the net amount of change in PD that occurred between the start of treatment and 9 months post-treatment (*P* > 0.05; Table 2).

**Table 1:** Mean probing depth, bone density, and root length between the groups at each time interval

Parameter	Time	Group 1 (CAOT without graft)		Group 2 (CAOT with xenograft)		Group 3 (CAOT with bioactive glass)		*P*-value
		Mean	SD	Mean	SD	Mean	SD	
Probing depth (mm)	T1	1.54	0.17	1.57	0.13	1.56	0.14	0.054
	T2	1.17^b^	0.16	1.19^a^	0.14	1.19^a^	0.17	0.047*
	T3	1.18^b^	0.19	1.20^a^	0.20	1.19^a,b^	0.18	0.012*
Bone density	T1	63.24^c^	7.21	65.54^b^	6.41	66.41^a^	6.32	<0.000*
	T2	33.42^c^	6.81	51.11^a^	6.21	42.32^b^	6.43	<0.000*
	T3	64.11^c^	6.22	97.53^a^	6.33	80.12^b^	6.31	<0.000*
Root length (mm)	T1	12.81^b^	0.73	12.82^a^	0.54	12.82^a^	0.51	0.037*
	T2	12.79	0.71	12.79	0.64	12.80	0.53	0.968
	T3	12.78	0.64	12.78	0.53	12.79	0.52	0.060

Notes: *P*, probability level; *P ≤ 0.05; different superscript letters in the same row are significantly different. *n* number; T1, before CAOT (baseline); T2, after debonding (post-treatment); T3, 9 months after debonding. *Abbreviations*: *CAOT* corticotomy-assisted orthodontic treatment, *SD* standard deviation

**Table 2:** Comparison of mean probing depth (mm) in each group at different time intervals

Group	Time interval	Mean difference	SD difference	*P*-value
Group 1	T1–T2	0.37	0.08	0.002*
	T1–T3	0.36	0.09	0.004*
Group 2	T1–T2	0.38	0.07	0.001*
	T1–T3	0.37	0.08	0.000*
Group 3	T1–T2	0.37	0.08	0.001*
	T1–T3	0.37	0.08	0.002*

Notes: *P*, probability level; *P ≤ 0.05; T1, before CAOT; T2, after debonding; T3, 9 months after debonding

*Abbreviation*: *SD* standard deviation

There was a statistically significant difference between the three groups regarding the change that occurred in PD during each time interval. Nine months post-treatment (during the retention period), group 1 demonstrated a mean PD of 1.18 ± 0.19 mm, while group 2 demonstrated a mean PD of 1.20 ± 0.20 mm and group 3 demonstrated a mean PD of 1.19 ± 0.18 (*P* > 0.05; Table 1).

#### Bone density

Within the three groups, there were significant differences in the amount of change that occurred in bone density during the different time intervals. During the period of active tooth movement (from pre-treatment to post-treatment), all groups demonstrated a decrease in bone density; the mean decrease was -29.82% in group 1, -14.43% in group 2, and -24.04% in group 3. The amount of increase in bone density from post-treatment to 9 months post-treatment was also significantly different between the three groups; the mean increase was 0.87% in group 1, 31.99% in group 2, and 13.71% in group 3 (Table 1). The net percentage increase in bone density from the start of treatment to 9 months post-treatment (97.53% ± 6.33%) was greater in group 2 than in groups 1 and 3.

#### Root resorption

There was no significant difference between the three groups in terms of the average root length values obtained pre-treatment, post-treatment, and 9 months post-treatment. The difference in the net amount of root resorption between the three groups was also statistically insignificant. The average net decrease in root length was -0.02 ± 0.02 mm in group 1, -0.03 ± 0.11 mm in group 2, and -0.01 ± 0.01 mm in group 3 (Table 1).

## Discussion

Most orthodontic patients, and particularly adults, are interested in the possibility of reducing their treatment time. Given this constant demand for shorter treatments, orthodontists have increasingly sought ways and new approaches to shorten the treatment duration without compromising results. CAOT has been suggested for reducing the orthodontic treatment time [5, 6, 8, 9, 28–30]. PAOO can play a major role in the comprehensive treatment of occlusal and esthetic needs in some patients. PAOO is an extension of previously described techniques involving surgical alteration of the alveolar bone to decrease treatment time [5].

In this study there was a significant reduction in the treatment time for adult patients demonstrating moderate crowding to an average of 11.4 (range 10 − 13) weeks. This is in agreement with other reports of CAOT [5–9, 30–32] and agrees with the findings of Wilcko et al., who presented cases treated over short periods with a combination of CAOT and periodontal alveolar augmentation [5, 8, 9, 27].

Thirty-three patients were initially enrolled in the present study, but four were excluded because of multiple missed appointments and failure to maintain good oral hygiene, when a considerable amount of patient cooperation was necessary. After 9 months, only 27 patients were available for clinical and radiographic re-evaluation; these patients had a good clinical outcome and did not experience any relapse.

Interestingly, the current results also showed that the total orthodontic treatment time was markedly reduced when compared with the average treatment time (31 months) for extraction therapy [2, 33]. In a clinical trial, Hajji [7] investigated the effects of resolving mandibular anterior dental crowding by comparing non-extraction, extraction, and CAOT approaches. The mean active treatment time for the CAOT group was 6.1 months versus 18.7 months in a non-extraction orthodontic group and 26.6 months in an extraction group, which is consistent with the findings of the present study.

Several theories have been proposed in an attempt to explain the rapid tooth movement observed after corticotomy. The initial concept, which prevailed in several subsequent reports, was based on “bony block movement”. According to this theory, the tooth embedded within a block of medullary bone serves as the handle by which the bands of less dense medullary bone surrounding the teeth are moved block by block [33]. However, the latest concept concerning the rapid tooth movement after corticotomy is supported by RAP, described as accelerated bone turnover and decreased regional bone density [8, 9, 12, 33, 34]. RAP is a local response to a stimulus, and describes a process by which tissue forms more rapidly than during the normal regional regeneration process. The term “regional” refers to the demineralisation of both the cut site and the adjacent bone and the term “accelerated” refers to an exaggerated or intensified bone response in cuts that extend to the marrow. It is explained as a temporary phase of localised soft and hard tissue remodelling that results in rebuilding of the injured sites to a normal state. By enhancing the various healing stages, this phenomenon causes healing to occur 2–10 times more rapidly than normal physiological healing. The RAP mechanism potentiating tissue healing has been shown to occur in the mandible as well as in the long bones [12, 34, 35].

No hazardous effects on the periodontium were observed after corticotomy in this study. PD in particular was not significantly different between the study groups (Table 1). Not only was there no deterioration in periodontal depth, but patients demonstrated a slight improvement in PD values.

Unlike most of the corticotomy procedures described earlier, the present cortical cuts were made in the labial cortical plate of bone only, without making subapical cuts, and the lingual flap was not reflected. This was performed to support the blood supply of the mandibular dentoalveolar region via the lingual mucosa and to protect the thin roots of the incisors in the region where access is difficult and there is a possibility of damaging the teeth. Labial subapical horizontal cuts were omitted to protect the overlying cortical bone and to maintain the blood supply to the incisors, because the spongiosa bone was not left intact. Furthermore, it was assumed that RAP induced by the buccal corticotomy would readily involve the non-corticotomised lingual side [28, 36]. Moreover, the main purposes of using the present conservative technique were to reduce the duration and extent of surgery and the postoperative discomfort by eliminating exposure of the patient to the risks of additional lingual surgery [28, 36]. In addition, using the current corticotomy technique, the vertical cuts were started 1 − 2 mm below the alveolar crest in an attempt to protect the crestal bone and periodontal membrane, as per previous recommendations [28].

Several reports have speculated that, by avoiding the crest of the marginal bone during vertical corticotomies, there would be less risk of damage to the marginal periodontium [5, 6, 28, 33, 37]. These surgical conservative criteria could explain the absence of detrimental clinical side effects on the periodontium in this study.

The current results are in accordance with other reports of rapid tooth movement and reduced treatment times without clinically noticeable adverse periodontal effects [8, 30, 38]. The fact that the teeth can be moved more rapidly, thus resulting in shortened treatment times, is certainly advantageous for periodontal health, because a reduction of the time that the patient needs a fixed appliance reduces patient “burnout” and substantially reduces the time available for relatively benign commensal bacterial biofilms to undergo qualitative changes and convert to a destructive cytotoxic (“periodontopathic”) type, which is often seen when fixed appliances have remained on the teeth for more than a year [9].

The present study revealed a reduction in alveolar bone density at the time of debonding (T2) in all three groups (Table 1). This is in agreement with other reports that have attributed this to the trauma caused to cortical bone, which has been shown to be a potentiating factor in producing localised osteoporosis or reversible osteopenia. For bone, this temporary condition means increased mobilisation of calcium, decreased bone density, and increased bone turnover, all of which would facilitate more rapid tooth movement [28, 39–41]. After an initial reduction, the bone density had increased again at 9 months post-treatment in all three groups (Table 2), which adds further support to the view that the dynamics of tooth movement in these patients might be more appropriately described as a demineralisation/remineralisation process, rather than bony block movement [8, 9, 15, 16, 28, 36, 39–41].

PAOO with bone augmentation was performed in the present study to increase alveolar volume and to avoid the possible risks of the procedure [8, 9]. It was considered that the roots would still have sufficient support if very large expansions were implemented to resolve severe crowding. The reshaped alveolar bone provides additional support for the roots of the teeth after completion of treatment and diminishes potential dehiscence [8, 9, 36]. Two different types of bone grafts, a xenograft (bovine xenograft) and an alloplast (bioactive glass), were utilised in this study. Numerous studies have shown the biocompatibility and effectiveness of a variety of xenografts that incorporate biomaterials derived from bovine bone. Other studies have reached the conclusion that this material has a system of interconnected pores, the structure of which is similar to that of human spongy bone, which may contribute to promoting the close interrelationship between hydroxyapatite and bone during growth [17–25]. Bioactive glass was selected as the second bone graft material because a number of in vivo and in vitro studies have highlighted its potential as a regenerative scaffold [18, 21, 22]. Moreover, highly bioactive glass particles have good clinical manageability and certain haemostatic properties, and showed both osteoproduction and osteoconduction [22].

It is interesting to note that, at the end of the study period, there was a significantly greater increase in bone density in the two groups that had been treated with bone grafting when compared with the group that had been treated with a modified CAOT alone (Table 1). Moreover, patients who were treated with the bovine-derived xenograft showed a greater (albeit not statistically significant) increase in bone density than those who were treated with bioactive glass. This could be attributed to a different ability of the two biomaterials to promote bone formation. In addition, the composition of animal bone is morphologically more similar than any synthetic product to human bone; a previous analysis of the results of clinical testing and the clinical take-up of different products developed by the biomedical industry has shown the overall superiority of bone substitutes of natural origin over derivative substitutes [40, 42].

In terms of root resorption, the current study revealed no significant difference in root length values between the three study groups (Table 1). Root resorption is a complex process, involving the combination of a multitude of biological and mechanical factors. It is generally accepted that some root resorption is expected with any orthodontic tooth movement [43] Thus, it is believed that the absence of any significant root resorption in this study might be, in part, attributed to the short treatment duration, which is in agreement with previous reports of this advantage of CAOT [22, 29, 31, 32, 44]. However, incorporation of bone grafting appears to have little effect on the incidence or amount of root resorption because of the absence of any statistically significant difference between the three groups regarding the amount of root resorption (Table 1).

## Conclusions

The results of this study suggest that PAOO is an effective and promising treatment approach that can decrease the active treatment duration, the risk of root resorption, and/or adverse periodontal effects in adult patients. PAOO may improve the state of the periodontium by increasing bone density and decreasing the risk of root resorption. Use of bovine-derived xenografts provided better results than bioactive glass in terms of increasing bone density. However, further randomised testing in humans is required to shed more light on an expanded use of PAOO, as well as to confirm the claimed advantages of this technique and evaluate the long-term effects and stability of such treatment.

## Abbreviations

CAOT: Corticotomy-assisted orthodontics
PAOO: Periodontally accelerated osteogenic orthodontics
PD: Probing depth
RAP: Regional acceleratory phenomenon
